# MicroRNA as a Novel Modulator in Head and Neck Squamous Carcinoma

**DOI:** 10.1155/2010/135632

**Published:** 2011-02-15

**Authors:** Li-Hsin Chen, Kun-Ling Tsai, Yi-Wei Chen, Cheng-Chia Yu, Kuo-Wei Chang, Shi-Hwa Chiou, Hung-Hai Ku, Pen-Yuan Chu, Ling-Ming Tseng, Pin-I Huang, Wen-Liang Lo

**Affiliations:** ^1^Institute of Pharmacology, National Yang-Ming University, Taipei 11221, Taiwan; ^2^Department of Medical Research and Education, Taipei Veterans General Hospital, Taipei 11217, Taiwan; ^3^Institute of Clinical Medicine, School of Medicine, National Yang-Ming University, Taipei 11221, Taiwan; ^4^Institute of Oral Biology and Biomaterial Science, Chung Shan Medical University, Taichung 40201, Taiwan; ^5^Department of Dentistry, School of Dentistry, National Yang-Ming University, Taipei 11221, Taiwan; ^6^Division of Oral and Maxillofacial Surgery, Department of Stomatology, Taipei Veterans General Hospital, Taipei 11217, Taiwan; ^7^Institute of Anatomy and Cell Biology, National Yang-Ming University, Taipei 11221, Taiwan; ^8^School of Medicine, National Yang-Ming University, Taipei 11221, Taiwan; ^9^Otolaryngology & Head and Neck Surgery, Department of Otolaryngology, Taipei Veterans General Hospital, Taipei 11217, Taiwan; ^10^Department of Surgery, Taipei Veterans General Hospital, Taipei 11217, Taiwan; ^11^Cancer Center, Taipei Veterans General Hospital, Taipei 11217, Taiwan

## Abstract

MicroRNAs have emerged as important regulators of cell proliferation, development, cancer formation, stress responses, cell death, and other physiological conditions in the past decade. On the other hand, head and neck cancer is one of the top ten most common cancers worldwide. Recent advances in microRNAs have revealed their prominent role in regulating gene expression and provided new aspects of applications in diagnosis, prognosis, and therapeutic strategies in head and neck squamous carcinoma. In the present paper, we focus on microRNAs showing significant differences between normal and tumor cells or between cells with differential ability of metastasis. We also emphasize specific microRNAs that could modulate tumor cell properties, such as apoptosis, metastasis, and proliferation. These microRNAs possess the potential to be applied on clinical therapy in the future.

## 1. Introduction

Head and neck squamous cell carcinomas (HNSCCs) account for approximately 10,000 emerging patients in the USA per year. In the past 5 years, new subjects for HNSCC in the USA increased approximately 25%, while the total initiated cancer cases only increased about 5% during the same period [1–6]. In Taiwan, HNSCC has been one of the 10 leading causes of cancer deaths in the past decades. The mortality rate also increased significantly, from 4.25 per 100,000 in 1995 to 9.6 per 100 000 in 2006, a 2.26-fold increase in one decade [7]. Several risk factors for HNSCC have been reported, such as oropharynx-related problems, betel quid chewing, cigarette, and alcohol abuse [7–11]. Betel quid chewing is widespread in Taiwan, especially among the indigenous people and blue-collar workers, with a total estimated two million habitual users (10% of population) [12]. Betel quid chewing has also been associated with cancer prognosis in a dose- and time-dependent fashion. In a study of 378 HNSCCC patients, the 5-year survival rate of betel quid chewers was significantly lower than that of nonchewers [13]. Areca nut extract (ANE) is highly cytotoxic and genotoxic to cultured human oral mucosal epithelial cells and fibroblasts. Exposure of human keratinocytes to ANE results in apoptosis, generation of reactive oxygen species, genetic damage, and micronuclei formation [14]. The same study has found that a 24-hour treatment with ANE induced mutations at the hypoxanthine phosphoribosyltransferase (HPRT) locus in human keratinocytes [14]. Increased intracellular levels of reactive oxygen species and 8-hydroxyguanosine in cells exposed to ANE have been also reported [14]. Arecoline has been shown to induce structural chromosomal aberration, sister chromatid exchange, and micronuclei formation in different cell types [15, 16]. Moreover, it has been shown that arecoline induced a significant elevation of cyclin-dependent kinase inhibitor 1 (p21/waf1) and a decline of cell division control protein 2 (cdc2) and cyclin B1 in gingival keratinocytes [17]. Despite the recent development of molecular-targeted therapeutics and other updated treatment regimens, HNSCC remains difficult to manage. Most patients with advanced HNSCC die from locoregional progression, with local recurrence rate up to 40% even after multimodality treatment [8, 18, 19], indicating that a better understanding of molecular pathogenesis for this disease is urgently needed.

Several molecular pathways are involved in the process of HNSCC carcinogenesis. For instance, high expression of cyclin A and cyclin D1 raises the carcinogenicity in oral cancers. Besides, p27^Kip1^, an inhibitor of cyclin-dependent kinases, has been reported as a prediction marker for survival rate [20–22]. As a tumor suppressor, p53 function is suppressed when overexpresing murine double minute gene 2 (MDM2) in oral cancer cells [23]. Moreover, many apoptosis-related, adhesion molecular-related, and inflammation-related proteins, such as activating protein-1 (AP1), survivin, Ras and Ras-related C3 botulinum toxin substrate 1 (Rac-1), cyclooxygenase-2 (COX-2), epidermal growth factor receptor (EGFR), human epidermal growth factor receptor 2 (Her-2), signal transducer, and activator of transcription 1 (STAT-1) as well as caspase-1, play important roles in HNSCC carcinogenesis [24–28]. 

MicroRNAs (miRNAs), approximately 18–25 nucleotides in length, are a group of endogenous small and noncoding RNAs. Since its first discovery in 1993, the biological function and biogenesis of miRNAs became popular topics for biomedical researches. They are transcribed to form a primary miRNA via RNA polymerase II. Primary miRNA is processed into precursor miRNA (pre-miRNA) by Drosha and DRG 8 and is then exported from nucleus to cytoplasm through exportin 5. The pre-miRNA is modified by Dicer and the matured miRNA forms. There are about 1000 different miRNAs which have been discovered and estimated in human genome [29]. MiRNAs play a critical role in the regulation of gene expression. As expected, miRNAs expression is highly correlated with human diseases, such as cancer and aging [30]. MiRNAs donot only act as oncogenes but also as tumor suppressors, which further implicates their role as therapeutic targets [31].

The aim of this paper is to highlight miRNA as a novel modulator in HNSCC. Considering the rising importance of miRNA and increasing cases of HNSCC, we hope this paper would shed some light on cancer therapy and provoke ideas for new research in order to find out more details about and mechanisms beneficial for therapeutics of HNSCC.

## 2. MiRNA-Related Ageing and Cancers

Epigenetics of ageing is an emerging field involving the mechanisms that affect gene expressions other than inherited DNA sequences regulating the tumorigenesis in multiple cancers. Factors such as DNA methylation, histone modification, chromatin remodeling, the polycomb protein groups, and noncoding RNAs can all contribute to the broad variety of phenotypes of ageing. The category of small noncoding regulatory RNAs includes miRNAs, small interfering RNAs (siRNAs), PIWI-interacting RNAs (piRNAs), small nucleolar RNAs (snoRNAs), and small nuclear RNAs (snRNAs). Among these, we focused on a group of microRNAs and their impact on facets of organismal aging as well as cellular senescence. Most miRNA-mediated regulation occurs at the posttranslational level, primarily through its near-perfect or partial complementary fit against the coding region or 3^'^ untranslated region (UTR) of target mRNA, leading to translational repression and/or degradation, therefore, the regulation of gene expression. In rare cases, they may also promote translation [32, 33]. 

The roles of miRNAs in cancers have been extensively investigated in the past few years. The relevance of miRNAs in cancer was suggested by the changes in expression patterns [34] and recurrent amplification as well as deletion of miRNA genes in cancers. Calin et al. were the first to report the signature of 13 microRNAs capable of distinguishing between indolent and aggressive chronic lymphocytic leukemia (CLL) [35]. Since then, several miRNAs have emerged as candidate component of oncogenes and tumor-suppressor networks. The miRNAs aberrant expressions in cancers are summarized in Table 1. 

It has been demonstrated that miRNAs can function both as oncogenes and tumor suppressors in the development of cancers. Recently, the connection of miRNA biogenesis and tumor suppressor networks was elucidated. p53 is a well-known tumor suppressor regulating diverse physical responses to many cancer-related stress signals, which could be translated to affect cell proliferation, cell death, DNA repair, and angiogenesis. Efforts have unraveled the linkage between p53 and several miRNAs. Xi et al. found that miR-30a-5p, miR-181b, miR-372, let-7g, miR-26a, let-7b, miR-296, and miR30a-3p were upregulated by wt-p53, whereas miR-15b, miR-27a, miR200c, miR-191, miR-30c, miR-25, miR-107, miR-339, miR-125, miR-27b, miR-23a, and miR-10a were downregulated by wt-p53 in human colon carcinoma cell lines [36], suggesting that p53 plays a role in a wide range of miRNA expression. Another study analyzing 470 miRNAs found that 12 miRNAs were significantly affected by p53 [37]. Recent evidences indicated that miRNAs are directly regulated by p53. He et al. reported that miR-34a, miR-34b, and miR-34c reflected p53 status and the expression of these miRNAs was induced by DNA damage and oncogenic stress in a p53-dependent manner leading to apoptosis or cellular senescence [38]. These findings elucidated that miRNAs play crucial roles in the p53 tumor suppressor pathways.

## 3. MiRNAs Target Molecular Pathways in HNSCC

### 3.1. MiRNA Expression Profiles

MiRNA profiling in head and neck cancer has been reviewed by Liu et al. and Tran et al. [39, 40]. Recently reported HNSCC-related miRNAs expression profiling is summarized in Table 2. Some miRNAs show consistently altered expressions in different studies. For example, the upregulated expression of miR-21, miR-31, miR-18, and miR-221 has been reported in at least 2 different study series. Similarly, the expression of miR-133a, miR-133b, miR-125a, miR-138, miR-139, miR-200c, miR-26b, miR-302b, miR-302c, miR-342, miR-371, and miR-373, is consistently reported to be downregulated in HNSCC. Exception for certain miRNAs, all published miRNA profiling shared little in common. This may be due to various types of sample applied. Generally, the analysis of upregulation and downregulation of miRNA profiles were of consistence. However, there still were considerable variations between different researches. These discrepancies may be due to different sampling locations/cell lines (miR-9, 16, 23b, 29c, 31, 34b, 138, 221, 375, and 449), methods for performing the assays (miR-16, 29c, and 340), and scope of the microarrays (miR-16, 23b, 29c, 221, 340, 375, and 449). For example, the same miRNAs possessed opposite profiles between the squamous cell carcinomas and the nasopharyngeal carcinomas/pleomorphic adenomas. These manifestations might be contributed from different cell differentiations that were modulated by the same miRNAs under various circumstances. Sampling technique of the tumor tissue also influenced the proportion of tumor cells to nontumor cells that may interfere with the sensitivity of real-time PCR and microarray analysis.

### 3.2. MiRNAs Affect Apoptosis or Metastasis

There are several reasons indicating that miRNAs play several roles in human cancer, one of which is miRNAs-mediated cell death. Apoptosis is the active process of programmed cell death. It happens in multicellular organisms and is controlled by an intracellular program of gene expression. In humans, the balance between proliferation and apoptosis is important and essential for homeostasis maintenance. Tumorigenicity would be relatively easy to take place when this homeostasis collapses. MiRNAs mediate tumorigenicity by directly targeting antiapoptotic genes expression or proapoptosis genes expression. 

Metastasis, on the other hand, is another specific property of cancer cells. MiRNAs directly influence metastasis and have great impact on prognosis in clinical evaluation. Many studies to be discussed below in this paper showed that miRNAs inhibition or overexpression affected both apoptosis and metastasis. However, it is difficult to tell if the results of migration or invasion were impacted by their effects on apoptosis despite that some miRNAs have been proved to directly target genes involved in metastatic capability.  Table 3 lists all miRNAs discussed below.

#### 3.2.1. Potential Tumor Suppressors

Several miRNAs are found to act as tumor suppressors. Hong et al. reported that inhibition of miR-296 expression could promote esophageal squamous cell carcinoma apoptosis. MiR-296 could up-regulate Bcl-2-associated X protein (Bax) expression, down-regulate B-cell lymphoma 1 protein** (**Bcl) expression, suppress cyclin D1 and p27 expression, and promote the chemotherapeutic sensitivity [41]. Long et al. showed that up-regulation of let-7a promotes laryngeal cancer cell dysfunction by modulating proliferation, inhibiting metastasis, and inducing apoptosis [42]. Comparing to normal human laryngeal cells or nondifferentiated cells, let-7a miRNA expression level was highly suppressed. Furthermore, in the same study, let-7a affected RAS and c-Myc expression in protein level, thus mediated cell apoptotic genes and oncogenes expression. Reduction of miR-138, on the other hand, was found in highly metastatic cells. Enforced expression of miR-138 could mitigate invasion, lead to cell cycle arrest, and induce cell apoptosis [43]. However, it is hard to tell if miR-138 does affect the ability of invasion or it is simply impacted by raising the percentage of apoptosis. Recently, the same group further demonstrated the role of miR-138 in metastasis. They showed that miR-138 modulated migration and invasion through targeting Ras homolog gene family, member C (RhoC) and Rho-associated, coiled-coil containing protein kinase 2 (ROCK2). Inhibition of miR-138 caused elongated cell morphology, increased cell stress fiber generation, and enhanced cell migration as well as invasion [44]. Other miRNAs such as miR-204 and miR-34c also inhibit invasion. Lee et al. had shown that miR-204 is a newly targeting gene which is a suppressor of metastasis in head and neck cancer. Enhancement of miR-204 expression directly led to the reduction of proliferation, invasion, and migration in HNSCC cell lines and mitigated experimental lung organ metastasis in animal study [45]. They also demonstrated that overexpression of miR-204 is associated with early recurrence in those HNSCCs expressing EGFR-pathway signature. MiR-34c, on the contrary, functioned as tumor suppressor that targeted 3^'^UTR of c-Met and inhibited cell growth and invasion [46]. This suggested that downregulation of miR-34c may contribute to malignancy in human laryngeal carcinoma through a mechanism involving targeting of c-Met.

#### 3.2.2. Apoptotic Antagonists or Metastasis Stimulators

On the contrary, several miRNAs act as a mediator of apoptotic antagonist or promote metastasis. Zhang et al. demonstrated that miR-141 could influence cell cycle, apoptotic phenotype, cell migration and invasion by regulating Bromodomain-containing protein 3 (BRD3), Ubiquitin-associated protein 1 (UBAP1), c-Jun N-terminal kinases (JNK), serine/threonine protein kinase (Akt/PKB), and phosphatase and tensin homolog (PTEN) signaling transduction pathways in nasopharyngeal carcinoma. The study showed a highly positive correlation between miR-141 and c-Myc expression and an opposite expression pattern between miR-141 and short palate, lung, and nasal epithelium clone 1 (SPLUNC1) [47]. Lee et al. published that miR-373 plays a pivotal role in regulating large tumor suppressor that homolog 2 (LATS2), a tumor suppressor has been implicated in modulating cell cycle and inducing apoptosis [48, 49]. MiR-373 is therefore considered to be a proliferation stimulator and an apoptosis inhibitor in human esophageal cancer [50]. MiR-21-attenuated cancer cells provoke apoptosis via modulating anti-apoptotic proteins. MiR-21 is found to target programmed cell death 4 gene (PDCD4), tropomyosin 1 (TPM1), and PTEN to inhibit apoptosis, promote transformation, and enhance colony formation [7, 51–53]. MiR-21 inhibitor displayed a statistically significant enhance in cytochrome c release and apoptotic expression, indicating that mir-21 is an unequivocal oncogenic miRNA in HNSCC. Recently, we explored that CD44, aldehyde dehydrogenase 1 (ALDH1), and phosphorylated STAT3 (p-STAT3) were higher in high-grade HNSCCs and that triple positivity for CD44/ALDH1/p-STAT3 indicated a worse prognosis for HNSCC patients [54]. In this study, CD44^+^ALDH1^+^ cells isolated from seven HNSCC patients showed greater tumorigenicity, radioresistance, and high expression of stemness (Bmi-1/Oct-4/Nanog) and epithelial-mesenchymal transitional (Snail/Twist) genes as p-STAT3 level increased. Recent studies showed that mir-21 is a key determinant in IL-11/STAT3 anti-apoptotic signaling pathway [55] and STAT3 activation of miR-21 and miR-181b-1 via PTEN and cylindromatosis (CYLD) are part of the epigenetic switch linking inflammation to cancer [56].

Additionally, a correlation was found between miR-211 expression level and the cancer metastasis in human oral carcinoma. MiR-211 expression is higher in tumors with vascular invasion and is correlated with poor prognosis. Enforced miR-211 expression intensively increased migration, colony formation, and tumorigenicity in oral squamous cell carcinoma (OSCC) cell line [57]. miR-222, on the other hand, regulates metalloproteinase 1 (MMP1) and manganese superoxide dismutase 2 (SOD2) expression by targeting their 3^'^UTR [58]. The reasons of miR-222 inhibited invasion was through both cis- and trans-regulatory mechanisms [58].

### 3.3. Proliferation

The capability of proliferation is a vital element of cancer cells. MiRNAs could target and regulate genes involved in cell cycle control or proliferation stimulation, and therefore, play a key role in modulating the phenotypes of cancer cells. Despite the promotion of metastatic ability of miR-211, forced expression of miR-211 accompanied with enhanced proliferation [57]. However, the underlying mechanism is still unknown. On the other hand, cyclin D1 is frequently overexpressed in cancer cells. Jiang et al. focused on cyclin D1 and tried to identify specific miRNAs targeting its mRNA. They found that miR-503 could not only suppress luciferase activity in reporter assay but also reduce mRNA and the protein level of cyclin D1 in human HNSCC cell line, UMSCC10B [59]. Despite the fact that miR-503 could alter S-phase and cause inhibition of cell proliferation, the physiological significance of miR-503 in HNSCC is still lacking. Cyclin E, another cell cycle regulator, is regulated by miR-15a. Anti-miR-15a, therefore, promoted DNA synthesis [60]. MiR-184 was discovered by comparing 4 tongue carcinomas and paired normal tissues through TaqMan miRNA assays. MiR-184 inhibition leads to proliferation inhibition, c-Myc reduction, and apoptosis in three SCC cell lines [61]. However, the authors did not explain the reduction of c-Myc nor if apoptosis was direct or indirect consequence of miR-184 inhibition. Further examination is required to understand the downstream targets of miR-184. Due to unknown mechanism, the plasma level of miR-184 highly was correlated with tumor status and seems to be a potential biomarker for SCC. MiR-100 and miR-125b are downregulated in OSCC. Exogenous expression of these two miRNAs could reduce cell proliferation [62]. However, the study only provided potential targets affected by miR-100 and miR-125b mimicking transfection. The direct targets are still undetermined. While Ubiquitin-like protein SUMO-1 conjugating enzyme (Ubc9) is upregulated in breast, head and neck, and lung cancer specimens, it could also be regulated by miR-30e [63]. In *HeLa* cells, ectopic expression of miR-30e could negatively regulate Ubc9 expression and suppress cell growth. Whether miR-30e has the same effects in HNSCC cells still needs to be verified. Additionally, in response to genotoxic stress, altered miRNAs might be associated with stress responses and be responsible for drug resistance of tumor cells. In HNSCC, for instance, miR-203 targets DeltaNp63 in response to genotoxic stress [64]. Under ultraviolet light c (UVC) exposure, miR-203 was upregulated and correlated with DeltaNp63 decrement. MiR-203, therefore, can regulate DeltaNp63 expression and control cell survival upon genotoxic stress.

## 4. The Relationship between MiRNAs, Hypoxia, and Epithelial-Mesenchymal Transition and Cancer

Poor prognosis of HNSCC patients was found to related to hypoxia [65–67] and epithelial-mesenchymal transition (EMT) [68]. Hypoxia is a critical and major feature of cancer microenvironment. It could affect and determine many vital responses of tumor cells and account for protection toward radiotherapy and chemotherapy. Yang and Wu. found that the hypoxic response is mainly mediated by hypoxia inducible factor-1 (HIF-1) that promotes EMT through direct regulation of a basic-helix-loop-helix transcription factor (TWIST) expression. They concluded that coexpression of HIF-1*α*, TWIST, and Snail could be used as a prognostic marker in HNSCC patients [69]. Several miRNAs, such as miR-210, are found to be hypoxia inducible. Upon hypoxia, miR-210 expression is induced. The majority of target candidates of miR-210 are genes expressed under normoxia. When miR-210-overexpressed cell lines were xenotransplanted into the mice, the tumor volumes were lower than the parental control. It suggests the fact that miR-210 may participate in tumor initiation in response to hypoxia [70] and is a prognostic indicator in HNSCC [71]. Two other miRNAs are also associated with hypoxia. MiR-31 ablates expression of HIF and regulates hypoxia responses [72, 73]. Also, in hypoxia, the expression of miR-98 is elevated, and it targets high-mobility group AT-hook 2 (HMGA2), which sensitizes HNSCC to topoisomerase II inhibitor, doxorubicin [74].

Epithelial-mesenchymal transition is a process by which epithelial cells lose their polarity and are converted to a mesenchymal phenotype, which has been recently regarded as the critical event to induce morphogenetic changes during embryonic development, organ fibrosis, and tumor metastasis [75–78]. Phenotypic changes of EMT include the downregulation of epithelial markers (e.g., E-cadherin, desmoplakin and plakoglobin) and upregulation of mesenchymal markers (e.g., vimentin, fibronectin, and *α*-smooth muscle actin) [75–78]. Transcriptional factors including Snail, Slug, TWIST, Zeb1, Zeb2, and E47 were shown to induce EMT through the repression of E-cadherin, and they are perceived as EMT regulators [79–84]. Major causes of HNSCC-related deaths are cervical node and distant metastasis. EMT was demonstrated to be the major mechanism responsible for mediating invasiveness and metastasis of late-stage cancers. Dr. Yang and his colleagues explored that Nibrin (NBS1) overexpression upregulated the expression of an EMT regulator Snail and its downstream target matrix metalloproteinase-2 [85]. Recent evidence suggests that depletion of the population of cancer stem cells (CSCs) decreases cancer recurrence and metastasis [86]. Prince et al. further demonstrated that the purified CD44^+^ population of HNSCC cells possesses the properties of cancer stem cells [87]. Subpopulation of CD44 positive cells showed chemoresistant genes ATP-binding cassette subfamily B member 1 (ABCB1), ATP-binding cassette sub-family G member 2 (ABCG2), cytochrome P_450_2C8 (CYP2C8), and telomerase reverse transcriptase (TERT) [88]. Our previous work showed that ALDH1^+^-lineage plays a crucial role in maintaining self-renewal and cancer stem-like properties in HNSCC cells [89]. ALDH1^+^-lineage cells are shown to have EMT shifting and endogenously co-expressed snail. Furthermore, the knockdown of snail expression significantly decreased the expression of ALDH1, inhibited cancer stem-like properties, and blocked the tumorigenic abilities of CD44^+^CD24^−^ALDH1^+^cells [89]. We also explored the enriched OC-SLC which possesses the characteristics of both stem cells and malignant tumors [90]. Additionally, expression of stemness markers (Nanog/Oct-4/CD133) contradicts the survival prognosis of OSCC patients [90]. Our data showed that ALDH1^+^ cells from HNSCC displayed higher levels of Bmi-1, and we further found that Bmi-1-silenced ALDH1^+^ cells showed increased sensitivity to radiotherapy and lower abilities for tumor invasion, colony formation, and self-renewal. Survival analysis further demonstrated that the mean survival rate of mice with ALDH1^+^ tumors under radiation treatment was significantly improved by knock-down of Bmi-1 [91]. More recently, Song et al. showed that Bmi-1 could directly promote EMT and malignancy in nasopharyngeal carcinoma by regulating snail [92]. Furthermore, there is growing evidence of the crosstalk and correlation between stemness pathways, tumor progression, and metastasis. The functional and mechanical significance of the overexpressed biomolecular pathway in HNSCC-CSC, however, is still blurred and needs to be clarified. 

Several miRNAs were reported to be associated with CSC in hepatocellular carcinoma, pancreatic cancer, breast cancer, and brain tumors [93–96]. It has been reported that overexpression of miR34 impairs the self-renewal properties of CSC isolated from brain tumors and pancreatic cancer [94, 97]. Tumorigenicity of breast CSC is also suppressed by ectopic let-7, miR200c, and miR30 expression [98–100]. MiR-181 family members are highly expressed in liver CSC, and silencing of miR-181 may eradicate liver cancer [93]. Therefore, it is of interest to ask whether microRNA is involved in regulating self-renewal and metastatic properties in HNSCC or HNSCC-associated CSC. In HNSCC, multiple miRNAs have been reported to be involved in HNSCC pathogenesis. It has been found that expression of oncogenic miR21 and miR184 promotes tumorigenicity in HNSCC [51, 61]. In contrast, ectopic expression of tumor-suppressive miR98, miR-137, and miR-193a resulted in a loss of cell growth of HNSCC. Recent clinicopathological findings demonstrated that the down-regulation of miR-133a, miR-133b, miR-205, and let-7d in HNSCC tumor tissues could be utilized for predicting the prognosis of patients with HNSCC [101, 102]. Our previous study also demonstrated that oncogenic miR31 and miR221 upregulated HIF expression and elevated tumorigenicity in HNSCC [57, 73].

MicroRNAs can act not only as oncogenes but also as tumour suppressors [29, 30]. Therefore, it is plausible to consider miRNAs as therapeutic targets in cancer cells [31]. MiR200c is a crucial modulator of EMT, tumourigenicity, and metastasis [99, 103–105]. It has been reported that miR200c is down-regulated and exhibits tumour-suppressive properties in renal cell, prostate, breast, bladder, pancreatic, and gastric cancers [106–110]. MiR200c is required for the growth of mammary stem cells and self-renewal properties of breast CSC through the direct targeting of Bmi-1 [99]. Down-regulation of miR-200c promotes EMT of breast cancer cells while overexpression of miR-200c induces mesenchymal-epithelial transition [105, 111]. Notably, miR-200c was reported to regulate pancreatic cancer cells, EMT, and cancer stemness properties by targeting ZEB1/ZEB2, Bmi-1, and Sox2 [112]. However, the role of miR200c in regulating tumourigenicity and metastasis in HNSCC or HNSCC-CSC has not been reported yet. Our recent study showed that the expression of miR200c in the regional metastatic lymph node of HNSCC tissues was significantly decreased, but Bmi-1 expression was increased as compared to parental tumours (unpublished data). Importantly, the site-directed mutagenesis with luciferase reporter assay showed that miR200c is targeting 3nesis with parental tumours (unpublished data). MiR200cs were significantly down-regulated while Bmi-1 was increased in HNSCC-ALDH1^+^/CD44^+^ compared to the other subsets of HNSCC cells. Notably, overexpressing miR200c further down-regulated the protein expressions of ZEB1, snail, and N-cadherin but up-regulated E-cadherin expression in ALDH1^+^/CD44^+^ cells. Xenotransplanted study confirmed that overexpression of miR200c or knockdown of Bmi-1 effectively inhibited the lung metastatic ability and prolonged the survival rate of ALDH1^+^/CD44^+^-transplanted mice.

## 5. Implications of MiRNAs as Biomarkers in HNSCC

Traditional tumor suppressor or proto-oncogene has been expended to the field of miRNA due to fast progress made in the past decade. Moreover, widespread technology of microarray provides fast and large-scale screening for potential targets in searching appropriate biomarkers. In HNSCC, a number of studies published potential biomarkers for cancer progression as well as prognosis. Even though contradiction among these studies was found, a new era has been opened up for therapeutic strategies in HNSCC. MiR-21 is frequently overexpressed in HNSCC. It is the most consistent miRNA found to be upregulated in many studies discussing miRNA profiling in HNSCC [61, 102, 113–115]. Nevertheless, the universal upregulation of miR-21 does not make it an appropriate marker for prognosis. Different conclusions were made in several studies. Cervigne et al. indicated that miR-21 along with miR-181b, and miR-345 are markers for oral cancer progression [116], and Li et al. suggested miR-21 as an indicator for poor prognosis in tongue SCC [51]. No significant result was obtained in other researches [71, 102]. A list of miRNA as potential prognostic marker was provided in Table 4.

The significance of polymorphisms at miRNA and miRNA target sites in disease risk and prognosis has been addressed and investigated [117, 118]. Because miRNAs regulate gene expression by binding to 3^'^UTR sites, this binding can be affected by single nucleotide polymorphism (SNP) resides within miRNA target sites. MiRNA or miRNA target site polymorphism could abolish the existing target sites or create illegitimate binding sites. These SNPs can therefore affect protein expression and lead to altered organismal phenotypes. According to Saunders et al., a relatively low polymorphism was found in functional region of miRNA while many target sites are disrupted by high frequency of SNPs. In HNSCC, let7-binding site polymorphism at 3^'^UTR of V-Ki-ras2 Kirsten rat sarcoma viral oncogene homolog (KRAS) (KRAS-LCS6) and sequence polymorphism in miR196A2 are both associated with reduced survival in head and neck cancer [119, 120]. We recently first demonstrated that let-7a expression was significantly decreased but that Nanog/Oct4 expression was increased in HNSCC tissues as compared to adjacent normal cells (accepted and unpublished data). Expression of let-7a in recurrent HNSCC tissue and in regional metastatic lymph nodes of HNSCC patients was also significantly decreased, but Nanog/Oct4 expression was increased as compared to the expression levels in the parental tumours. Consistently, the stemness genes were significantly up-regulated, and let-7a was down-regulated in HNSCC-ALDH1^+^ cells relative to HNSCC-ALDH1^−^ cells. Furthermore, lentiviral-mediated let-7a overexpression could significantly inhibit the stemness signature and the chemoresistant abilities of HNSCC-ALDH1^+^ cells. Most importantly, overexpression of let-7 or knockdown of Nanog in ALDH1^+^ cells effectively blocked tumour metastasis and significantly prolonged survival time in ALDH1^+^-transplanted immunocompromised mice. Overall, restoration of let-7a in HNSCC and HNSCC-tumour initiating cells may be a new approach for the therapeutic treatment of HNSCC in the future.

## 6. Conclusions

Traditionally, the major theory of cancer is considered as dysregulation of protein-coding tumor suppressor genes and oncogenes. To date, the discovery of epigenetic regulation provides new explanations to and reveals a more complicated network of cancer formation. MiRNAs, a small group of noncoding RNAs, draw more attention than ever and are thought to be a new category of tumor suppressor or oncogene. Accumulating knowledge in miRNA brings new perspectives in understanding of cell transformation and tumorigenicity. Nevertheless, growing numbers of HNSCC cases need a breakthrough to improve the mortality rate of HNSCC patients. Further studies are needed to understand the mechanism of dysregulation of miRNA as well as their targets.

## Figures and Tables

**Table 1:** MicroRNA aberrant expression in cancer.

Tumor type	Upregulated miRNA	Downregulated miRNA	References
Glioblastoma	miR-21	miR-7	[121–125]
	miR-221		
	miR-222		
Lung	let7-5a	miR-1, let-7 family, miR-7,	[121, 126–130]
	miR-17-92 cluster, miR-18b,	miR-15a/miR-16, miR-29	
	miR-20a, miR-21, miR-34c,	family, miR-32, miR-137,	
	miR-106a, miR-155, miR-182,	miR-342	
	miR-328	miR-145	
Breast	miR-10b, miR-21, miR-22,	let-7, miR-7, miR-9-1,	[34, 131–133]
	miR-27a, miR-155, miR-210,	miR-17/miR-20, miR-31,	
	miR-221, miR-222, miR-328,	miR-125a, miR-125b,	
	miR-373, miR-520c	miR-146, miR-200 family,	
		miR-205, miR-206,	
		miR-335	
Hepatocellular carcinoma	miR-17-92 cluster, miR-21,	miR-1, miR-34a	[134–140]
	miR-143, miR-221	miR-101, miR-122a	
	miR-224	miR-125b	
Gastric cancer	miR-21, miR-27a, miR-130b	miR-101, miR-143, miR-145	[141–143]
Colorectal cancer	miR-17-92 cluster, miR-21	miR-34a, miR-34b/c, miR-127, miR-143,	[144–149]
		miR-145, miR-342	
Prostate cancer	miR-221, miR-222	miR-15a-miR-16-1 cluster,	[144, 150–153]
		miR-101, miR-127,	
		miR-449a	
Lymphoma	miR-17-92 cluster, miR-155	miR-143, miR-145	[154–156]
CLL	miR-21, miR-155	miR-15, miR-16, miR-29b,	[35, 156–160]
		miR-29c, miR-34a,	
		miR-143, miR-145,miR-181b, miR-223	

CLL: chronic lymphocytic lymphoma.

**Table 2:** Antecedent studies identified microRNA expression level change in head and neck cancer.

miRNA	Up-regulated	Down-regulated	Study groups/methods	References
let7i; 15a; 15b; 17a; 18; 18a; 18b; pre 21; 21; 21-17p; 24-1p; 98; 99b; 104; 126; 130b; 137; 140; 142-3p; 146; 146b; 151; 152; 155; 181b; 181d; 184; 188; 192; 193b; 199b-2p; pre-205; 213; 301; 325; 333; 337; 338; 374; 455; 491	**v**			[61, 102, 113–115, 161–167]

let-a family; let-7 family; let7a; 10b; 20b; 23 family; 27 family; 30; 34 family; 34c; 93-7p; 99a; 100; 107; 125; 125a; 127; 144; 154; 195; 200 family; 200a; 291-3p; 368; 370; 378; 422 family; 494		**v**		[61, 102, 113, 114, 119, 162, 164–168]

9	**v**		13 NPC/real time-PCR	[162]
		**v**	18 OC cell lines/real-time-PCR	[164]

16	v		9 HNSCC cell lines/Microarray	[113]
		**v**	31 NPC/real time-PCR	[166]

17	**v**		13 NPC/real time-PCR	[162]
		**v**	31 NPC/real time-PCR	[166]

23b	**v**		104 HNSCC/Microarray and real time-PCR	[102]
		**v**	5 SCC/real time-PCR	[165]

29c	**v**		4 HNSCC/Microarray	[114]
		**v**	31 NPC/real time-PCR	[166]

31	**v**		18 OC cell lines/real time-PCR	[164]
		**v**	13 NPC/real time-PCR	[162]

34b	**v**		5 SCC/real time-PCR	[165]
		**v**	13 NPC/real time-PCR	[162, 166]

31 NPC/real time-PCR
138	**v**		13 NPC/real time-PCR	[162]
		**v**	4 OSCC/real time-PCR	[61]

221	**v**		99 HNSCC and 14 normal/Microarray and real time-PCR	[115, 165]
			5 SCC/real time-PCR	
		**v**	31 NPC/real time-PCR	[166]

340	**v**		18 OC cell lines/real time-PCR	[164]
		**v**	9 HNSCC cell lines/Microarray	[113]

375	**v**		169 HNSCC/real time-PCR	[161]
		**v**	99 HNSCC and 14 normal/Microarray and real time-PCR	[115, 167]

16 pleomorphic adenoma/Microarray and real time-PCR
449	**v**		16 pleomorphic adenoma/Microarray and real time-PCR	[167]
		**v**	9 HNSCC cell lines/Microarray	[113]

MiRNA: microRNA; NPC: nasopharyngeal carcinoma; OC: oral cancer; HNSCC: head and neck squamous cell carcinoma; SCC: squamous cell carcinoma.

**Table 3:** miRNAs involved in tumorigenesis of HNSCC.

microRNA	Tumor sites or cell lines	Physiological effects	Potential target(s)	Reference(s)
let-7a	Laryngeal squamous cancer tissues; Hep-2 cells	Let-7a mimic transfection suppressed proliferation and induced apoptosis in Hep-2 cells	RAS and c-MYC	[42]
miR-10b	Human esophageal cancer cell lines (KYSE30, KYSE70, KYSE140, KYSE150, KYSE410, KYSE450, KYSE510, and EC9706)	Ectopic expression of miR-10b promoted migration and invasion	KLF4	[169]
miR-15a	HNSCC SQ20B cell line	miR-15a inhibition promoted DNA synthesis	Cyclin E	[60]
miR-21	Primary tongue carcinoma and primary esophageal squamous cell carcinomas; human tongue cancer cell lines (SCC-15 and CAL27) and esophageal squamous cell carcinoma cell lines (TE6, TE8, TE10, TE11, TE12, and TE14)	miR-21 inhibition inhibited cell growth and promoted apoptosis	TPM1, PTEN, and PDCD4	[51–53]
miR-31	OSCC SAS cell line	Ectopic expression increased the oncogenic potential of HNSCC cells under normoxia conditions	FIH	[73]
miR-100	UPCI:SCC029 cells	Transfection of miR-100 mimics inhibited proliferation	D1, EGR2, MMP13, and FGFR3	[62]
miR-125b	UPCI:SCC029 cells	Transfection of miR-125b mimics inhibited proliferation	KLF13, CXCL11, and FOXA1	[62]
miR-138	Six paired HNSCC cell lines (UM1/UM2, 1386Tu/1386Ln and 686Tu/686Ln with different migration or invasion ability	Ectopic expression caused cell cycle arrest, apoptosis, and suppressed invasion	RhoC and ROCK2	[43, 44]
miR-141	5–8F cell line (an NPC cell line) knockdowned c-MYC or re-expressed SPLUNC1	miR-141 inhibition decreased migration, inhibited invasion, and slightly enhanced apoptosis	BRD3, UBAP1, and PTEN	[47]
miR-184	TSCC Cal27, HN21B, and HN96 cell lines	miR-184 inhibition resulted in proliferation inhibition and apoptosis	c-Myc	[61]
miR-196	Esophageal cancer tissues	Downregulation of miR-196 inhibited growth and conferred drug-sensitivity of esophageal cancer cells	N.D.	[41]
miR-200c	5 primary HNSCC CSC	Upregulation of miR-200c inhibited the cancer stem cell-like properties of ALDH1^+^CD44^+^ HNSCC	Bmi-1	To be published
miR-203	HNSCC JHU-012 cells	miR-203 induced cell cycle arrest and apoptosis upon UVC irradiation	DeltaNp63	[64]
miR-204	HNSCC cell lines (SCC25, SCC35, SCC58, SCC61, SCC135, SCC151, SQ20B, SQ38, and JSQ3)	miR-204 mimics repressed proliferation, invasion, and migration	N.D.	[45]
miR-210	Pancreatic cancer cell line SU86.86 and Fadu cells	Ectopic expression repressed xenograft tumor growth	HOXA1, HOXA9, and FGFRL1	[70]
miR-211	OSCC SAS cell line	Enforced expression of miR-211 enhanced migration, invasion, and colony formation	N.D.	[57]
miR-222	HNSCC UM1/UM2 cell lines	Transfection of miR-222 mimics inhibited invasion	MMP1 and SOD2	[58]
miR-373	The esophageal cancer cell lines (CE48T, CE146T, KYSE70, KYSE150, KYSE170, KYSE5, 10KYSE50, and CE81T)	miR-373 enhances cell proliferation in esophageal cancer cell lines	LATS2	[50]
miR-503	UMSCC10B cell line	Transfection of miR-503 decreased S phase and cell growth	Cyclin D1	[59]

N.D: not determined.

Abbreviations: HNSCC: head and neck squamous cell carcinoma; OSCC: oral squamous cell carcinoma. NPC: nasopharyngeal carcinoma; TSCC: tongue squamous cell carcinoma; CSC: cancer stem cell.

**Table 4:** Potential miRNAs for prognosis in HNSCC.

Potential microRNA as prognostic marker	Type of abnormal expression	Type of samples	Reference(s)
miR-21		OSCC	
miR-181b	Overexpression		[116]
miR-345			
Combined Let-7d and miR-205	Underexpression	HNSCC	[102]
miR-21	Overexpression	TSCC	[51]
miR-210	Overexpression	HNSCC	[71]
miR-211	Overexpression	OSCC	[57]
miR-296	Overexpression	ESCC	[41]
Combined miR-221 and miR-375	Overexpression of miR-221	HNSCC	
			[115]
	Underexpression of miR-375		

OSCC: oral squamous carcinoma cell; HNSCC: head and neck squamous carcinoma cells; TSCC: tongue squamous carcinoma cell; ESCC: esophageal squamous carcinoma cells.
